# Neuroendocrine Tumor Causing Carcinoid Syndrome in the Elderly

**DOI:** 10.7759/cureus.29736

**Published:** 2022-09-29

**Authors:** Asma U Hosna, Daniel Miller, Roger Stern, Deesha Shah

**Affiliations:** 1 Internal Medicine, Icahn School of Medicine at Mount Sinai, Queens Hospital Center, New York, USA

**Keywords:** geriatrics and internal medicine, gastroenterology, neuroendocrine, carcinoid syndrome, gastrointestinal carcinoid tumor

## Abstract

Neuroendocrine tumors (NETs) causing carcinoid syndrome are very rare. Patients can have very general symptoms and tumors can be missed especially in the elderly where many symptoms may be attributed to the aging process. It is of the utmost importance to do a full workup on elderly patients even if the symptoms can be explained by aging alone.

## Introduction

Neuroendocrine tumors (NETs) are rare neoplasms that arise from enterochromaffin cells and are usually found in the gastrointestinal tract or bronchopulmonary system. Clinical symptoms tend to be very generalized and are therefore often attributed to other conditions or just the aging process in general [1]. Symptoms largely depend on the size and location of the tumor. Tumors in the gastrointestinal tract tend to cause gastrointestinal symptoms such as diarrhea, while NETs in the lung can cause bronchospasms and difficulty breathing. We present a case where a 93-year-old male patient had carcinoid syndrome from a NET.

## Case presentation

A 93-year-old male with a past medical history of hypertension, hyperlipidemia, coronary artery disease status post coronary artery stenting, and hypothyroidism, and a past surgical history of a coronary artery bypass graft and a cholecystectomy, presents with a chief complaint of chronic, intractable watery diarrhea for three months. He reports approximately eight bowel movements a day. He often has a sudden urge to move his bowels immediately after starting to eat a meal, and he is particularly troubled by incontinence when he is unable to reach the bathroom fast enough. He has tried many over-the-counter remedies and several courses of antibiotics without relief. He denies any relieving factors. He endorses a 40-60 lbs weight loss in the past three months. He also endorses an unproductive cough during this time as well. He admits to a 40-pack-year smoking history but he states that he quit 20-years-ago. 

A physical exam revealed a well-nourished appearing man in no acute distress. Pupils were equal, round, and reactive to light. No tonsilar exudates were appreciated and no lymphadenopathy was observed. Auscultation of the heart revealed a normal rate and rhythm with normal heart sounds and no rubs, gallops, or murmurs. Lung sounds were diminished bilaterally. An abdominal exam revealed loose skin and a right upper quadrant scar consistent with a previous cholecystectomy. All four quadrants had frequent bowel sounds and were soft and non-tender to palpation. The patient was wearing a diaper filled with well-masticated food but looked undigested as some lettuce was easily noticeable in the stool. A digital rectum exam revealed a normal muscular tone of the external anal sphincter.

The patient was admitted with a differential list including pancreatic insufficiency, *Clostridium difficile*,* *bacterial and parasitic infection, vasoactive intestinal peptide tumor, and carcinoid syndrome. The fecal fat test and *Clostridium difficile* antigen screen were both negative. Stool culture and a stool test for ova and parasites were negative. The vasoactive intestinal peptide was within normal limits. Chromogranin A was elevated to 405.9 ng/ml (normal < 101.8 ng/ml). A 24-hour urine 5-hydroxyindoleacetic acid (5-HIAA) was elevated to 62.4 mg/24 hours (normal < 6 mg/24 hours).

**Figure 1:**
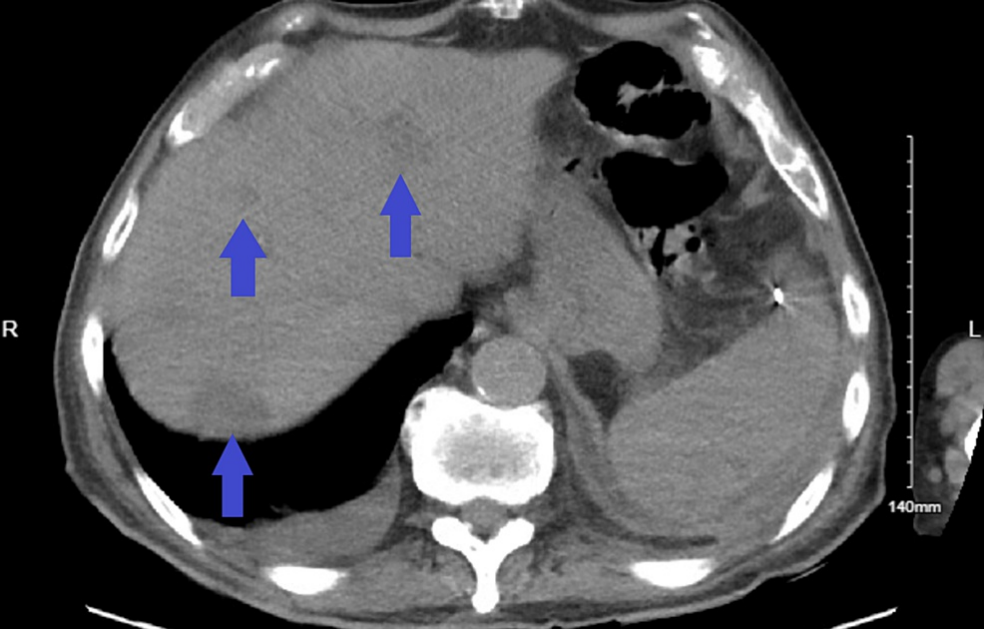
CT Abdomen Showing Multiple Lesions in the Right and Left Hepatic Lobes Blue arrows indicate the location of the lesions

**Figure 2:**
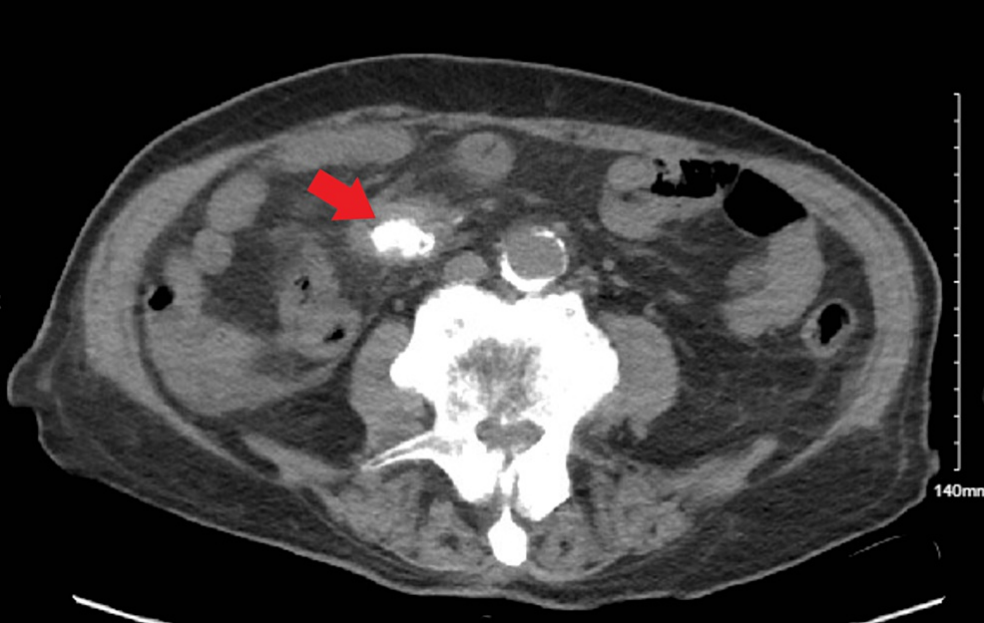
CT Abdomen Revealing a Mesenteric Calcified Mass Red arrow indicates the location of the mass

Abdominal computed tomography (CT) showed multiple lesions in the liver and spleen, as well as a calcified mesenteric mass, which was suggestive of a carcinoid tumor (Figures 1, 2).

The patient ultimately underwent a liver biopsy and the diagnosis of carcinoid tumor was confirmed. The patient was referred to oncology for further management.

## Discussion

Carcinoid tumors are the most commonly documented manifestation of gastrointestinal NETs. These tumors are most commonly thought of to have an indolent, asymptomatic presentation, however, it has been shown that these neoplasms often can exhibit a malignant clinical course. Typical symptoms depend on the location of the NET, as described in the introduction, precipitated by biologically active secretagogues. Carcinoid tumors have the capacity to secrete a menagerie of biologically active substances. The most commonly secreted peptide is serotonin. The release of serotonin into the systemic circulation can cause what is known to be carcinoid syndrome, which can include intractable diarrhea, flushing of the skin, broncho-constriction, and, later in the disease course, right-sided valvular heart disease. In the presence of localized disease, carcinoid tumors produce serotonin, which is sequestered in platelets while the remainder is metabolized in the liver and lung and transformed into 5-HIAA. In the context of liver metastases of carcinoid tumors, however, patients may develop the systemic symptoms of carcinoid syndrome, which is when these secretagogues produced by the carcinoid tumor escape the ability of the liver to metabolize and proceed to seed into the systemic circulation [2].

In a study that included a series of over 13,000 cases of carcinoid tumors reported to the Surveillance, Epidemiology, and End Results program with the National Cancer Institute, the incidence rates for caucasian men and women were 2.47 and 2.58 per 100,000 individuals. Statistics were somewhat higher for black men and women (4.48 and 3.98 per 100,000 individuals) [3].

The diagnosis of suspected carcinoid disease is typically first investigated by measuring serotonin metabolites in the patient's urine, namely, 5-HIAA, which can be quantified in a 24-hour urine sample. This modality has been found to have high specificity but has lower sensitivity. Once the biochemical diagnosis of carcinoid disease is established, imaging studies can be performed to establish tumor localization. Abdominal CT has been the standard means of imaging to localize carcinoid tumors and potential metastatic disease. The sensitivity of abdominal CT is poor, however, the use of radiolabeling and scintigraphy offers increased information in patients with carcinoid tumors if abdominal CT is insufficient and there is a high clinical suspicion of carcinoid syndrome. Effective treatment of carcinoid disease includes surgical resection of the primary tumor, debulking, and medical therapy for symptomatic relief. Specific treatments are detailed and individualized to the case, the location of the primary tumor, and the presence of metastases, the standard for treatment of carcinoid tumors remains surgical intervention [4].

## Conclusions

Elderly patients with concerning symptoms should be worked up as opposed to just attributing the symptoms to the aging process. Serious conditions can be missed if a comprehensive examination is not performed. It is imperative to view symptoms independent of a person's age and only after should you weigh the risks and benefits before doing any diagnostic tests.
